# Neurodevelopment and neural environment inform Alzheimer's disease age at onset and phenotype

**DOI:** 10.1002/alz.70668

**Published:** 2025-09-17

**Authors:** Zachary A. Miller, Rik Ossenkoppele, Neill R. Graff‐Radford, Isabel E. Allen, Wendy Shwe, Lynne Rosenberg, Dustin J. Olguin, Michael G. Erkkinen, P. Monroe Butler, Salvatore Spina, Jennifer S. Yokoyama, Rahul S. Desikan, Philip Scheltens, Wiesje van der Flier, Yolande Pijnenburg, Emma Wolters, Rosa Rademakers, Daniel H. Geschwind, Joel H. Kramer, Howard J. Rosen, Katherine P. Rankin, Lea T. Grinberg, William W. Seeley, Virginia Sturm, David C. Perry, Bruce L. Miller, Gil D. Rabinovici, Maria Luisa Gorno‐Tempini

**Affiliations:** ^1^ Memory and Aging Center Department of Neurology UCSF Weill Institute for Neurosciences University of California San Francisco California USA; ^2^ Dyslexia Center Department of Neurology and Psychiatry UCSF Weill Institute for Neurosciences University of California San Francisco California USA; ^3^ Alzheimer Center Amsterdam Department of Neurology Amsterdam Neuroscience Vrije Universiteit Amsterdam Amsterdam UMC Amsterdam the Netherlands; ^4^ Clinical Memory Research Unit Skånes universitetssjukhus VE Minnessjukdomar Lund University Malmö Sweden; ^5^ Department of Neurology Mayo Clinic Jacksonville Florida USA; ^6^ Department of Biostatistics University of California San Francisco San Francisco California USA; ^7^ School of Medicine George Washington University Washington District of Columbia USA; ^8^ Department of Medicine Pediatrics University of Colorado Boulder Colorado USA; ^9^ Department of Neurology Brigham and Women's Hospital Harvard Medical School Boston Massachusetts USA; ^10^ Department of Radiology and Biomedical Imaging University of California San Francisco San Francisco California USA; ^11^ Department of Applied and Translational Neurogenomics VIB Center for Molecular Neurology Wilrijk Belgium; ^12^ Department of Neurology David Geffen School of Medicine University of California Los Angeles Los Angeles USA; ^13^ Department of Pathology University of California San Francisco San Francisco California USA

**Keywords:** alzheimer's disease, autoimmune disease, early‐onset alzheimer's disease, heterogeneity, learning disability, logopenic variant primary progressive aphasia, non‐amnestic alzheimer's disease, non–right‐handedness, posterior cortical atrophy, seizure

## Abstract

**INTRODUCTION:**

Risk factors associated with sporadic non‐amnestic and early‐onset Alzheimer's disease (AD) remain underexamined.

**METHODS:**

We investigated a large, clinically heterogeneous, AD cohort for frequencies of established risk factors (hypertension, hypercholesterolemia, diabetes mellitus) alongside novel factors (non–right‐handedness, learning disability, seizures, autoimmune disease).

**RESULTS:**

Early‐onset AD possessed lower frequencies of established risk factors (hypertension, hypercholesterolemia, diabetes mellitus, all *p *< 0.001) and higher frequencies of novel factors (non–right‐handedness, learning disability, active seizure, all *p *< 0.001; remote seizure, *p* = 0.002; and autoimmune disease, *p *= 0.007). An age at onset < 70 maximally distinguished novel from typical factors. Principal component analysis loaded novel factors into two components, non–right‐handedness and learning disability versus seizure and autoimmune disease, which combined, resulted in an exponential decrease in age at onset from one factor alone.

**DISCUSSION:**

Identifying novel factors enriched in early‐onset and non‐amnestic AD introduces new theories of AD susceptibility and phenotypic heterogeneity, with significant implications for disease prediction and treatment.

**Highlights:**

- We identified a suite of novel factors overrepresented in early‐onset and non‐amnestic AD.
- These factors can be broadly conceptualized as neurodevelopmental (non–right‐handedness and learning disability) and neural environmental (seizure and autoimmunity).
- The combination of these factors produced exponential decreases in AD age at onset, compared to each alone, supporting a new theoretical framework for understanding AD risk with implications for disease prediction, prevention, and therapeutic intervention.

## 1 BACKGROUND

Alzheimer's disease (AD) is the largest contributor to dementia world‐wide. With a rapidly aging population, lacking powerful interventions, the world faces a health‐care crisis. To date, mitigating the effects of established AD risk factors like hypertension, hypercholesterolemia, diabetes mellitus, and limited schooling, through advancements in the treatment of vascular disease and improved access to education, is beginning to have an impact on reducing the global burden of AD.^1^ As established AD‐associated risk factors come almost exclusively from studies of typical amnestic late‐onset AD (LOAD; age at first symptoms of ≥ 65) and are largely age‐related, we sought to identify novel AD associated risk factors by examining the demographic and health histories of individuals presenting with early‐onset AD (EOAD).

The distinction between EOAD and LOAD is historic and largely arbitrary, based on the misconception that declines in cognitive status at or after age 65 reflected a distinctly different process than declines witnessed prior to age 65.^2^ Nevertheless, meaningful differences between early and late presentations of AD exist. EOAD is less common than LOAD, accounting for 4% to 10% of all AD. Typically, AD is a memory‐predominant, hippocampal‐based amnestic syndrome, but EOAD cases demonstrate a greater variety of phenotypes that include behavioral, dysexecutive, apraxic, visuospatial, or focal language AD clinical presentations. Together, these variants are referred to as atypical focal cortical or non‐amnestic AD. Non‐amnestic AD presentations are also more common within EOAD, constituting up to 25% to 50% of EOAD compared to < 10% of LOAD. Furthermore, the most common genetic risk factor for AD, apolipoprotein E (*APOE*) ɛ4, is less common in EOAD, most notably so in non‐amnestic presentations.^3^ Despite sharing the same underlying pathological substrate as LOAD, the reasons for the earlier age at onset, greater degree of clinical heterogeneity, and decreased frequency of *APOE* ɛ4 in EOAD remains poorly understood.

Recognizing that neurodegenerative disorders start focally, spread in a network‐based fashion, and produce distinctive clinical phenotypes,^4^ we identified that neurodevelopmental differences (non–right‐handedness and learning disability)^5^, ^6^ and neural environmental insults (seizures, chronic increased systemic inflammation, and history of autoimmune disease)^7^, ^8^ were associated with early‐onset and/or non‐amnestic forms of dementia (primary progressive aphasias). In the present study, we investigated these same neurodevelopmental and neural environmental factors in a large heterogenous AD cohort, to determine whether they influence symptom age at onset and the phenotypic presentation of AD.

## 2 METHODS

### 2.1 Discovery cohort

**FIGURE 1 alz70668-fig-0001:**
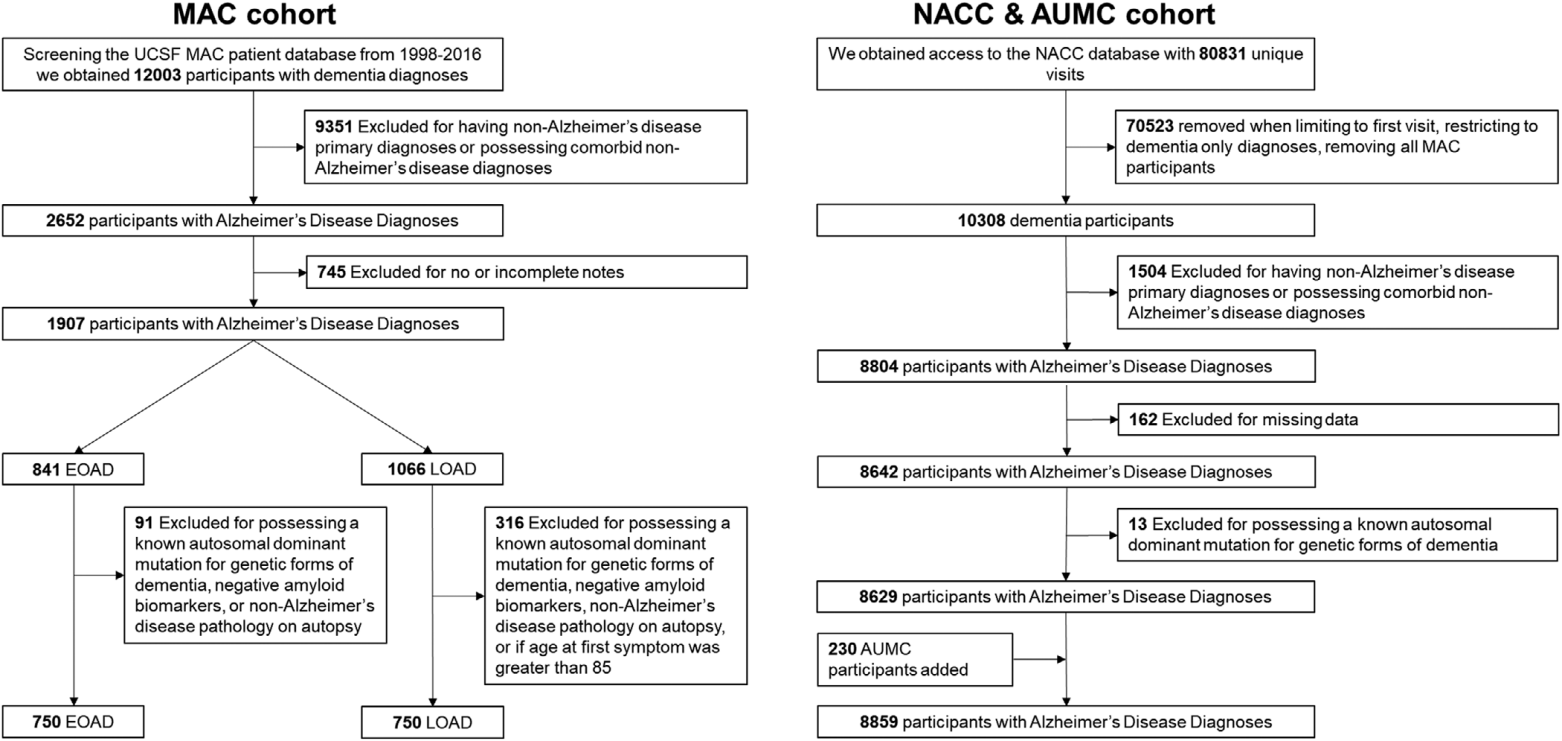
MAC cohort and NACC & AUMC cohort flowchart. As our intention was to study individuals with sole predicted underlying AD pathology, subjects were excluded if they possessed (1) clinical diagnoses of comorbid Lewy body, Parkinson's disease, and/or significant evidence of vascular disease defined by subcortical ischemic vascular dementia criteria^13^; (2) incomplete charts (defined as missing age at first symptoms, hand preference, past medical history, and/or social history); (3) known autosomal dominant AD and/or FTD mutations (*APP, C9ORF72, FUS, GRN, MAPT, PSEN1*, and *PSEN2*), and or diagnoses of Down syndrome; (4) when available, were negative for AD biomarkers (CSF, Amyvid, and/or PiB PET) and/or AD pathology at autopsy;^63^ or (5) were older than age 85 at first symptoms, as the epidemiology is understudied but believed to differ from typical amnestic AD, possessing a greater amount of multiple neurodegenerative disease co‐pathologies.^14^, ^15^ AD, Alzheimer's disease; AUMC, Amsterdam University Medical Center; CSF, cerebrospinal fluid; EOAD, early‐onset Alzheimer's disease; FTD, frontotemporal dementia; LOAD, late‐onset Alzheimer's disease; MAC, Memory and Aging Center; NACC, National Alzheimer's Coordinating Center; PET, positron emission tomography; PiB, Pittsburgh compound B.

Searching the University of California San Francisco (UCSF) Memory and Aging Center (MAC) database, we identified *n* = 2652 subjects from 1998 to 2016 who met diagnostic (National Institute of Neurological and Communicative Disorders and Stroke–Alzheimer's Disease and Related Disorders Association and later National Institute on Aging–Alzheimer's Association) criteria for probable AD.^9^, ^10^ EOAD and LOAD status were determined based on the age at first symptom with EOAD defined as < 65 and LOAD ≥ 65. Non‐amnestic AD classifications were restricted to diagnoses of logopenic variant primary progressive aphasia (lvPPA)^11^ or posterior cortical atrophy (PCA),^12^ given the degree of AD clinicopathological correlation. Individuals were excluded if they possessed clinical diagnoses of comorbid Lewy body, Parkinson's disease, and/or significant evidence of vascular disease defined by subcortical ischemic vascular dementia criteria^13^ or if they possessed incomplete charts (defined as missing age at first symptoms, hand preference, past medical history, and/or social history). Also excluded were subjects in whom amyloid biomarker data or an autopsy pathology report was inconsistent with a diagnosis of underlying AD, or if an individual possessed a known autosomal dominant genetic cause for dementia, even if that gene was a known cause of AD, as the goal of this study was to investigate factors that impact idiopathic forms of disease. We excluded cases whose age at onset was greater than age 85 as knowledge about AD epidemiology in populations older than 85 is limited and begins to demonstrate much greater amounts of co‐pathology, suggesting that this group does not reflect the sample underlying biology as typical LOAD.^14^, ^15^ Splitting into EOAD and LOAD cohorts, we included 750 consecutive cases of EOAD and then case matched by number, LOAD cases (Figure 1). A subset of cases (*n* = 273 total) had undergone autopsy and/or obtained amyloid beta (Aβ) positron emission tomography (PET) imaging.

### 2.2 External validation cohorts

Selecting individuals meeting criteria for dementia with an etiology of AD from the National Alzheimer's Coordinating Center (NACC) database,^16^, ^17^ filtering out MAC cases, incomplete charts, and known autosomal dominant disease‐causing mutations, produced 8629 subjects from September 2005 to June 2016. As the NACC database lacked sufficient numbers of non‐amnestic AD, we obtained 230 additional subjects from Amsterdam University Medical Center (AUMC; 36 lvPPA, 61 PCA, 66 EOAD, 67 LOAD), increasing the total to 8859 (Figure 1). For comparison of autoimmune prevalence, specifically, we supplemented the AUMC group with cases from the Mayo Clinic, Jacksonville (MCJ; 43 lvPPA, 81 PCA). The MCJ participants were not incorporated in the above 8859 collection, to avoid the possibility of co‐enrollment in the NACC.

### 2.3 Identification and classification of typical AD risk and novel factors

At each site, charts were reviewed, screening for hypertension, hypercholesterolemia, diabetes, handedness, learning disability,^5^ and seizure (as defined by the NACC: “active” if the event occurred within the past year or still required active management, “remote” if the event occurred > 1 year ago and completely resolved or is without ongoing management), and autoimmune disease^5^, ^18^ (Table S1 in supporting information).

**RESEARCH IN CONTEXT**

1. **Systematic review**: Research is lacking on the demographic and health history factors relevant to early‐onset and atypical forms of Alzheimer's disease (AD). Papers were identified through PubMed searches. Relevant articles’ bibliographies were further screened.
2. **Interpretation**: In this case–control study of > 10,000 AD patients, we identified a series of novel factors overrepresented in early‐onset and non‐amnestic disease (non–right‐handedness, learning disability, seizure, and autoimmunity). Principal component analysis reduced these factors into two components we labelled “neurodevelopmental” differences and “neural environment” insults, the combination of which produced exponential decreases in AD age at onset, compared to each alone.
3. **Future directions**: The identified differences in demographic and health history between early onset and atypical AD from late‐onset AD support a new theoretical framework for understanding AD risk, with implications for predicting and preventing neurodegenerative disease. Multicenter investigations, integrating collection of these novel factors, across healthy aging and dementia cohorts, are urgently needed.

### 2.4 Data analyses

Data[Fig alz70668-fig-0001] were summarized for EOAD and LOAD groups using means and standard deviations for continuous variables and counts and proportions for categorical data. For comparisons between EOAD and LOAD, analysis of variance or Student independent groups *t* tests were used for continuous variables such as age and education and Fisher exact or chi‐squared tests for nominal variables such as sex, non–right‐handedness, learning disability, or presence of autoimmune disease. Odds ratios between EOAD and LOAD for risk factors were calculated using logistic regression controlled for significant covariates. For validation of the groupings of these risk factors within EOAD and LOAD, principal component analysis identified composite risk factors by EOAD and LOAD using the Kaiser criterion (principal component eigenvalues > 1.0) with loadings > 0.4 or < –0.4 as cutoffs used to identify variable importance in each identified factor. In addition, we created heatmaps using the Krakov visual techniques and odds ratios to identify differences and similarities between the cohorts.^19^ The heatmaps were compared visually to identify similarities and differences and using chi‐squared tests to test for independence. All analyses were performed with STATA 17.1 with significance levels set to *p *< 0.05. Although the quantitative analyses involved many combinations of outcomes and predictors, we did not perform formal adjustments for multiple comparisons for each of the factors. This was because (1) the hypotheses were highly specific and (2) we expected many measures to show statistically significant differences between groups and the directions and magnitudes of the differences could fit a biologically coherent pattern with each result reinforcing the other, rather than detracting from one another, as required by formal multiple comparison adjustments such as Bonferroni.^20^, ^21^

## 3 RESULTS

Within the MAC cohort, EOAD showed significantly higher proportions of non–right‐handedness, learning disability, seizure, autoimmune disease, and non‐amnestic AD presentations, while LOAD demonstrated higher frequencies of hypertension, hypercholesterolemia, and diabetes (*p *< 0.001 for all except remote seizure, *p *= 0.002, and autoimmune disease, *p *= 0.007). Sex, education, and *APOE* ɛ4 allelic frequencies were no different between groups. External cohorts, from the NACC and AUMC replicated increased non–right‐handedness and seizure within EOAD and increased proportions of hypertension, hypercholesterolemia, and diabetes associated with LOAD (*p *< 0.001 for all comparisons except handedness, *p *= 0.02). NACC and AUMC LOAD showed a higher proportion of women, fewer years of education, and decreased *APOE* ɛ4 allelic frequencies compared to EOAD (*p *< 0.001 for all comparisons; Table 1). To confirm these results, we performed logistic regressions predicting EOAD versus LOAD controlling for sex, education, *APOE* ɛ4 status, and typical and novel risk factors, which confirmed all significant analyses (Table 2).

**TABLE 1:** Demographics and typical and novel factors in MAC and NACC and AUMC in EOAD versus LOAD.

	MAC 1500 AD			NACC & AUMC		
Group demographics	EOAD (*n* = 750)	LOAD (*n* = 750)	*p*	EOAD (*n* = 2274)	LOAD (*n* = 6585)	*p*
**Age at onset** Average years ± SD	**55.8 ± 5.5**	**71.5 ± 4.2**	**<0.001**	**57.1 ± 5.7**	**74.9 ± 6.1**	**<0.001**
**Typical risk factors**						
**Age at first visit** Average years ± SD	**60.2 ± 5.9**	**75.0 ± 4.3**	**<0.001**	**62.4 ± 6.7**	**79.5 ± 6.2**	**<0.001**
**Sex** % Male (*n*)	41.9% (314/750)	44.1% (331/750)	n.s.	**47.3% (1075/2274)**	**42.4% (2789/6585)**	**<0.001**
**Education** Average years ± SD	15.2 ± 3.6 (728)	15.1 ± 3.8 (729)	n.s.	**14.3 ± 3.7 (2255)**	**13.7 ± 3.9 (6542)**	**<0.001**
***APOE* ɛ4 carriers** % with one or more ɛ4 alleles	**52.5% (156/297)**	**63.0% (102/162)**	**0.03**	60.8% (1060/1742)	58.3% (2683/4605)	n.s.
**Hypertension** % Hypertension (*n*)	**34.9% (262/750)**	**54.7% (410/750)**	**<0.001**	**36.4% (826/2269)**	**58.5% (3843/6566)**	**<0.001**
**Hypercholesterolemia** % Hypercholesterolemia (*n*)	**42.5% (319/750)**	**54.1% (406/750)**	**<0.001**	**43.2% (974/2254)**	**51.9% (3375/6507)**	**<0.001**
**Diabetes** % Diabetes mellitus (*n*)	**8.0% (60/750)**	**13.9% (104/750)**	**<0.001**	**9.8% (221/2266)**	**14.3% (943/6572)**	**<0.001**
**Novel factors**						
**Non–right‐handed** % non–right‐handed (*n*)	**15.5% (116/750)**	**8.4% (63/750)**	**<0.001**	**9.4% (214/2265)**	**7.9% (520/6575)**	**0.02**
**Learning disability** % learning disability (*n*)	**11.1% (83/750)**	**2.5% (18/750)**	**<0.001**			
**Remote seizure** % seizure (*n*)	**3.6% (27/750)**	**1.2% (9/750)**	**0.002**	**2.4% (52/2206)**	**1.2% (75/6486)**	**<0.001**
**Active seizure** % seizure (*n*)	**7.1% (53/750)**	**3.1% (23/750)**	**<0.001**	**2.4% (54/2260)**	**1.1% (73/6559)**	**<0.001**
**Autoimmune disease** % autoimmunity (*n*)	**25.5% (191/750)**	**19.6% (147/750)**	**0.007**			
**Non‐amnestic AD** % non‐amnestic AD (*n*)	**26.0% (195/750)**	**11.9% (89/750)**	**<0.001**	**4.2% (96/2274)**	**0.6% (38/6585)**	**<0.001**
**Amyloid confirmed** % w/+ autopsy and/or PET (*n*)	**27.5% (206/750)**	**8.9% (67/750)**	**<0.001**			

Abbreviations: AD, Alzheimer's disease; *APOE*, apolipoprotein E; AUMC, Amsterdam University Medical Center; EOAD, early‐onset Alzheimer's disease; LOAD, late‐onset Alzheimer's disease; MAC, Memory and Aging Center; NACC, National Alzheimer's Coordinating Center; SD, standard deviation.

Bold values shows statistical results.

**TABLE 2:** Logistic regressions of factors in MAC and NACC and AUMC that predict EOAD versus LOAD.

	MAC 1500 AD				NACC & AUMC			
	95% CI for OR				95% CI for OR			
	OR	Lower	Upper	*p*	OR	Lower	Upper	*p*
**Education**	1.022	0.953	1.095	n.s.	0.882	0.774	1.004	n.s.
***APOE* ɛ4 carriers**	0.674	0.437	1.038	n.s.	1.109	0.508	2.422	n.s.
**Decreased EOAD prevalence**								
Hypertension	**0.445**	**0.362**	**0.548**	**<0.001**	**0.405**	**0.367**	**0.446**	**<0.001**
Hypercholesterolemia	**0.627**	**0.511**	**0.836**	**<0.001**	**0.706**	**0.641**	**0.777**	**<0.001**
Diabetes mellitus	**0.540**	**0.386**	**0.755**	**<0.001**	**0.643**	**0.551**	**0.751**	**<0.001**
**Increased EOAD prevalence**								
Non–right‐handed	**2.029**	**1.464**	**2.816**	**<0.001**	**1.218**	**1.031**	**1.439**	**0.020**
Learning disability	**5.051**	**3.012**	**8.547**	**<0.001**				
Remote seizure	**2.677**	**1.150**	**5.759**	**0.012**	**2.183**	**1.506**	**3.163**	**<0.001**
Active seizure	**1.953**	**1.150**	**3.315**	**0.013**	**2.315**	**1.506**	**3.163**	**<0.001**
Autoimmune disease	**1.587**	**1.064**	**2.364**	**0.023**				

Abbreviations: AD, Alzheimer's disease; *APOE*, apolipoprotein E; AUMC, Amsterdam University Medical Center; CI, confidence interval; EOAD, early‐onset Alzheimer's disease; LOAD, late‐onset Alzheimer's disease; MAC, Memory and Aging Center; NACC, National Alzheimer's Coordinating Center; OR, odds ratio.

Bold values shows statistical results.

To determine whether these results were mediated by the increased proportion of non‐amnestic AD within EOAD, we separated the non‐amnestic AD cases from the total 1500 AD cohort, and all prior EOAD and LOAD differences survived. Comparing the non‐amnestic AD cohort against the remaining amnestic EOAD and amnestic LOAD revealed statistically lower *APOE* ɛ4 allelic frequencies in non‐amnestic AD (*p *< 0.001), while rates of *APOE* ɛ4 were no different between amnestic EOAD and amnestic LOAD (Table S2 in supporting information). As a further sub‐analysis, we split the non‐amnestic AD cohort into its constituent groups, lvPPA and PCA. The PCA cohort was significantly younger at age at onset and age of first visit, possessed a lower frequency of diabetes, and higher rates of active seizure and autoimmune disease (Table S3 in supporting information).

As these groups were based on the clinical diagnosis of an AD syndrome, we performed analyses within each of the diagnoses (non‐amnestic AD, amnestic EOAD, amnestic LOAD) broken down by cases with and without confirmed Aβ disease (autopsy proven and/or amyloid biomarker PET imaging positivity) to determine how well our results generalized to pathologically proven AD. Non‐amnestic AD and amnestic EOAD amyloid‐confirmed cases were younger at age at onset and age of first visit than their unconfirmed counterparts. Consistent with this younger age, they also possessed relative decreases in vascular risk factors. There was a greater proportion of male participants in amyloid‐confirmed amnestic EOAD and LOAD groups than those without amyloid confirmation, and across all diagnoses, years of education was higher in the amyloid‐confirmed group. There were no statistically significant differences among the novel factors between amyloid‐confirmed and unconfirmed groups, except for an even greater amount of learning disability within the amyloid‐confirmed non‐amnestic AD (Table S4 in supporting information).

**FIGURE 2:**
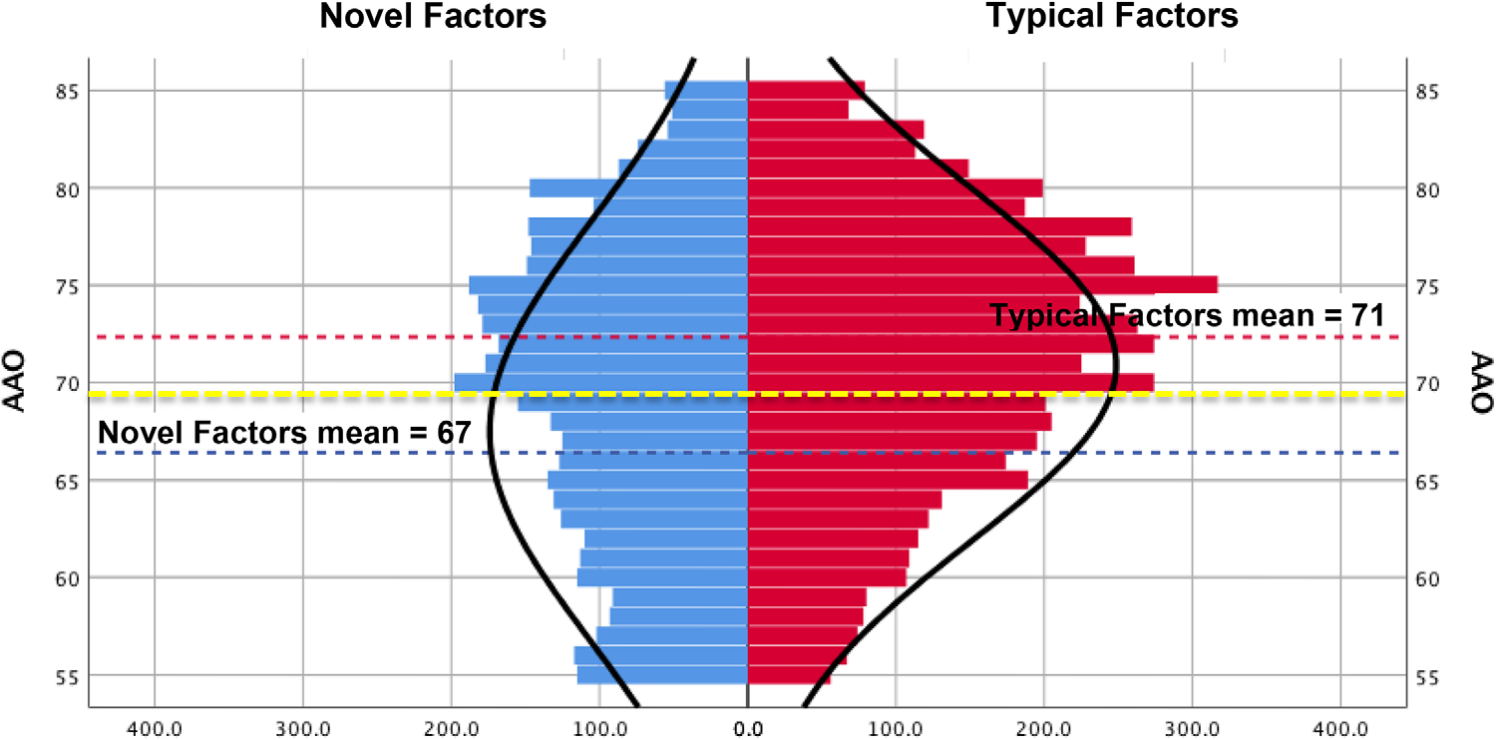
Histograms of Age at Onset by Novel vs. Typical Factors. Plotting the MAC, NACC, and AUMC individuals who possessed novel factors (only non–right‐handedness and seizure were assessed, as these were the only novel factors consistently collected across the various cohorts) in blue, and typical AD risk factors (hypertension, hypercholesterolemia, and diabetes mellitus) on the right, in red, by 2‐year AAO epochs, we obtained distribution curves for each. The mean AAO for novel factors was 66.9 years (blue dashed line), for typical factors, 71.4 years (red dashed line), and these distributions differed statistically (*p *= 0.003). Taking the mean of these two means, 69.2 years (yellow dashed line), maximally distinguishes the amounts of novel factors versus typical AD risk factors within this cohort. AAO, age at onset; AD, Alzheimer's disease; AUMC, Amsterdam University Medical Center; MAC, Memory and Aging Center; NACC, National Alzheimer's Coordinating Center.

Reducing the novel and typical risk factors into single composites (positive for typical factors if hypertension, hypercholesterolemia, and/or diabetes were present, and for novel factors only if non–right‐handedness and/or seizure were present; as autoimmune disease and learning disability were not adequately collected in the NAAC, these categories were not considered in this analysis), within a combined MAC/NACC and AUMC cohort, histogram plots revealed a mean age at onset of 66.9 years for novel factors, 71.4 years for typical AD risk factors, and 69.2 as the average difference between these two (Figure 2).

To determine how novel factors interacted with the most significant AD risk factor, *APOE* ɛ4, we isolated the subset of individuals who were positive for a novel factor and had undergone *APOE* ɛ4 testing, stratifying by *APOE* ɛ4 positive and negative status. Further, as rates of *APOE* ɛ4 differed in non‐amnestic AD cohorts from the remaining EOAD and LOAD, we plotted the non‐amnestic AD cohort separately. Here, we found that within the non‐amnestic AD cohort, there were no age at onset differences between *APOE* ɛ4 positive carriers and negative carriers, whereas in the remaining EOAD and LOAD, the interaction of *APOE* ɛ4 with novel factors was significant (*p *= 0.013), with *APOE* ɛ4 positive carriers showing first symptoms on average 3 years before those who were *APOE* ɛ4 negative (Figure S1 in supporting information).

**FIGURE 3:**
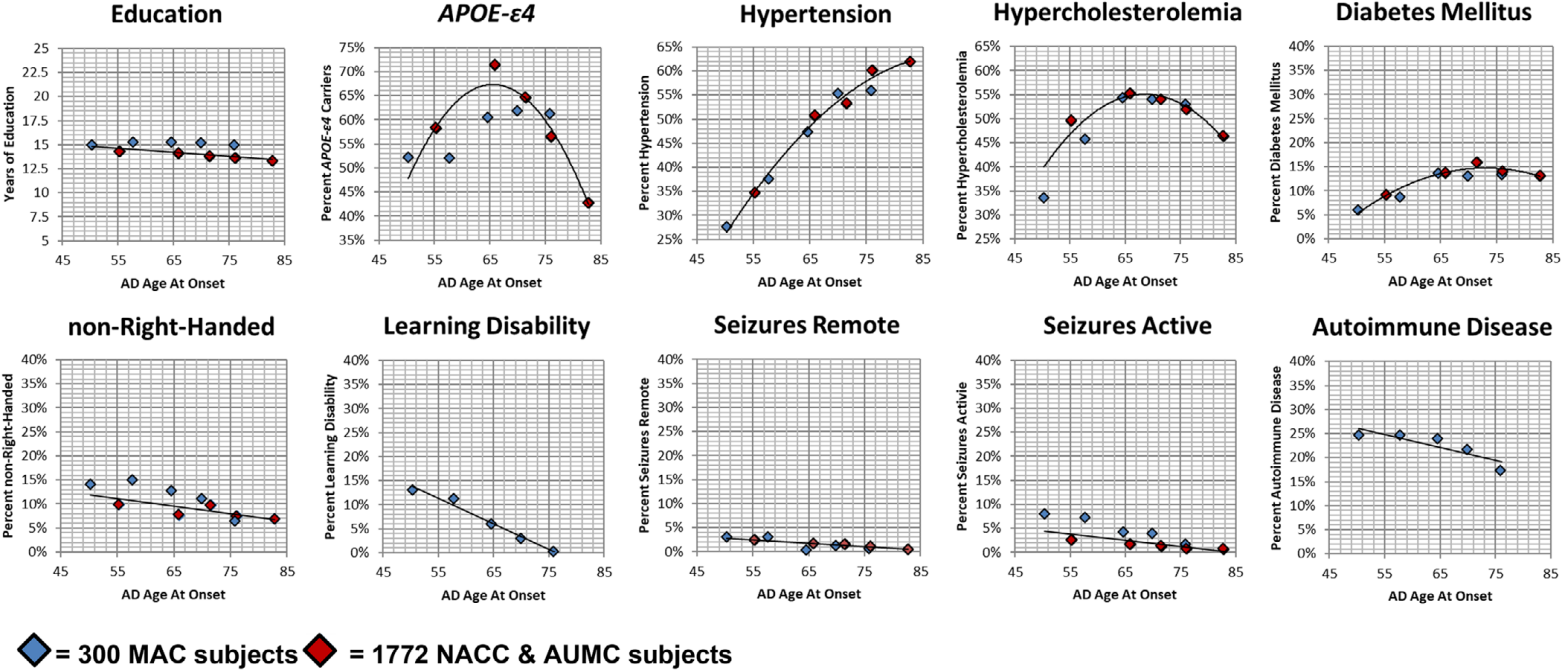
MAC 1,500 AD and NACC & AUMC AD Split into Quintiles. MAC and NACC & AUMC AD distribution of factors across age at onset split into quintiles. Each diamond represents a quintile with its respective cohort, with each blue diamond reflecting the 300 MAC subjects and each red diamond, the NACC & AUMC cohort. Each diamond is plotted out with the average of that specific cohort by the percent of a specific factor, like non–right‐handedness. The exact numerical values for each quintile can be found in supplemental material (Table S5 in supporting information). Accounting for weighted differences between the red and blue diamonds, best‐fit lines were generated. The top row consists of factors already known to impact AD risk, typical AD risk factors, while the bottom row reflects the series of novel factors we investigated in this study. AD, Alzheimer's disease; *APOE*, apolipoprotein E; AUMC, Amsterdam University Medical Center; MAC, Memory and Aging Center; NACC, National Alzheimer's Coordinating Center.

As age at onset determination is inherently imprecise (based on clinician report of the patient's or informant's testimony), we split each cohort into quintiles to investigate how various factors displayed across age at onset. Each quintile of the MAC cohort consisted of 300 individuals (rank ordered by the youngest to oldest age of age at onset) and each quintile of the NACC was made up of 1772 individuals (rank ordered from youngest to oldest cohort), save the final quintile that consisted of 1771 cases. The quintiles were graphed based on the average age of onset for each by the average percent of the factor present. Best‐fit lines were generated accounting for weighted differences between the MAC and NACC quintiles. With this, novel factors followed inverse linear relationships with age at onset, while the typical factors mostly fit quadratic trends (Figure 3 and Table S5 in supporting information).

**FIGURE 4:**
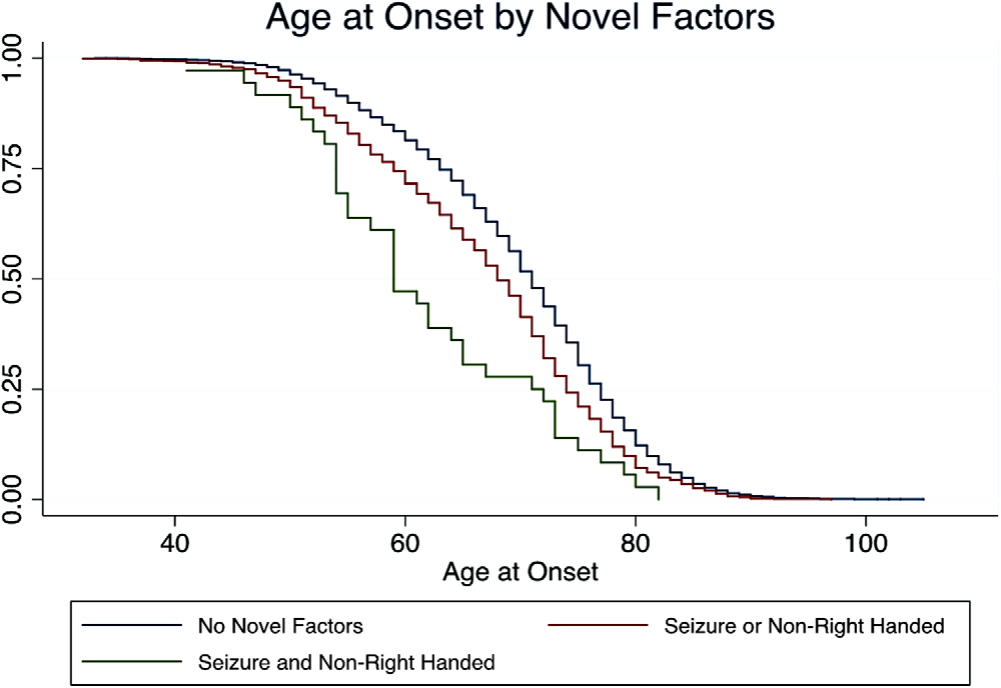
Burden of Factors on AAO. Burden of factors seizure and non–right‐handedness on AD age at onset. In combined MAC and NACC and AUMC cohorts, plotting individuals stratified by numbers of novel factors (non–right‐handedness and seizure) versus the age at which they developed first symptoms produced three distinct Kaplan–Meier curves. The blue line (*n* = 9074) encompasses all participants who lacked any novel factor. The red line (*n* = 1190) included only those who had one novel factor (either non–right‐handedness or seizure). The green line (*n* = 36) comprised those individuals who possessed both novel factors (non–right‐handedness and seizure). The age at which 50% of individuals with no novel factors develops first symptoms is 71, with only one factor is 68, and with both factors is 59. Using analysis of variance with a Scheffe correction for multiple comparisons: no novel factors versus one novel factor (blue vs. red) *p* < 0.001; no novel factors versus two novel factors (blue vs. green) log rank *p* < 0.001; one novel factor versus two novel factors (red vs. green) *p* = 0.009. AD, Alzheimer's disease; AUMC, Amsterdam University Medical Center; MAC, Memory and Aging Center; NACC, National Alzheimer's Coordinating Center.

To determine the degree of impact each novel factor yielded within the AD MAC cohort, we divided the MAC cohort by individual factor of interest and found that non–right‐handedness, learning disability, active seizure, remote seizure, and autoimmune groups, were individually significantly younger at age at onset (*p *< 0.001 for all, except autoimmune disease, *p *< 0.05; Table S6 in supporting information). Principal component analysis loaded all factors into three components, one consisting of the typical AD risk factors (hypertension, hypercholesterolemia, and diabetes) and two separating novel factors non–right‐handedness and learning disability from autoimmune disease and seizure. As offered above, within the NACC collection, among the novel factors, only non–right‐handedness and seizure history were adequately captured. As non–right‐handedness and seizure reflected factors within each of the two components identified in the MAC cohort, we combined the MAC and NACC and AUMC cohorts, restricting analyses to non–right‐handedness and seizures. Survival curve analysis demonstrated that the age at which 50% of those with only one novel factor (non–right‐handedness or seizure) developed first AD symptoms was 3 years younger than those without either factor (68 vs. 71). The group with both novel factors (non–right‐handedness and seizure) was 9 years younger than those with only one single novel factor (59 vs. 68) and 12 years younger than those with neither (59 vs. 71; log‐rank *p *< 0.001 across all comparisons using analysis of variance with a Scheffe correction for multiple comparisons; Figures 4 and S2).

To display the relative contributions of autoimmunity within the various AD cohorts, we created heat maps reflecting the prevalence of individual autoimmune conditions compared to general population values. Autoimmune conditions displayed a gradient of prevalence in which both the number of distinct autoimmune disorders as well as the degree of their overrepresentation was greatest in non‐amnestic AD, followed by EOAD, and least in LOAD. Within the MAC cohort, six of the seven top overrepresented autoimmune conditions (greater than five times general population estimates) were also identified within the AUMC and MCJ cohort, at near identical elevated rates (Table 3). In support of this qualitative assessment, formal chi‐squared analysis of the top six conditions in the MAC cohort and within the AUMC and MCJ cohort was not significant, indicating they are not independent (*p *= 0.544).

**TABLE 3:** Autoimmune prevalence and estimated odds ratios in MAC and AUMC & MCJ across total AD cohort and subtypes.

	MAC 1500 AD cohort					AUMC & MCJ			
	Total	Non‐amnestic	EOAD	LOAD	General Population	Total	Non‐amnestic	EOAD	LOAD
Autoimmune disease	(*n* = 1500)	(*n* = 284)	(*n* = 555)	(*n* = 661)	Prevalence	(*n* = 354)	(*n* = 221)	(*n* = 66)	(*n* = 67)
**ANCA‐associated vasculitis**	**23**	**n/a**	**60**	**n/a**		**93**	**n/a**	**505**	**n/a**
(prevalence)	0.07%	0.00%	0.18%	n/a	0.003%[48]	0.28%	0.00%	1.50%	n/a
(*n*)	1	0	1	0		1	0	1	0
**Guillain‐Barré syndrome**	**17**	**53**	**18**	**n/a**		**14**	**23**	**n/a**	**n/a**
(prevalence)	0.33%	1.06%	0.36%	n/a	0.02%[49]	0.28%	0.45%	n/a	n/a
(*n*)	5	3	2	0		1	1	0	0
**Systemic lupus Erythematosus**	**14**	**18**	**n/a**	**23**		**28**	**45**	**n/a**	**n/a**
(prevalence)	0.27%	0.35%	n/a	0.45%	0.02%[50]	0.56%	0.90%	n/a	n/a
(*n*)	4	1	0	3		2	2	0	0
**Idiopathic thrombocytopenic purpura**	**13**	**35**	**18**	**n/a**		**28**	**45**	**n/a**	**n/a**
(prevalence)	0.13%	0.35%	0.18%	n/a	0.01%[51]	0.28%	0.45%	n/a	n/a
(*n*)	2	1	1	0		1	1	0	0
**Multiple sclerosis**	**9**	**29**	**6**	**3**		**9**	**15**	**n/a**	**n/a**
(prevalence)	0.53%	1.76%	0.36%	0.15%	0.06%[50]	0.56%	0.90%	n/a	n/a
(*n*)	8	5	2	1		2	2	0	0
**Sarcoidosis**	**7**	**9**	**9**	**4**		**7**	**11**	**n/a**	**n/a**
(prevalence)	0.27%	0.35%	0.36%	0.15%	0.04%[52]	0.28%	0.45%	n/a	n/a
(*n*)	4	1	2	1		1	1	0	0
**Systemic sclerosis/scleroderma**	**6**	**15**	**8**	**n/a**		**n/a**	**n/a**	**n/a**	**n/a**
(prevalence)	0.13%	0.35%	0.18%	n/a	0.02%[53]	n/a	n/a	n/a	n/a
(*n*)	2	1	1	0		0	0	0	0
**Myasthenia gravis**	**3**	**18**	**n/a**	**n/a**		**n/a**	**n/a**	**n/a**	**n/a**
(prevalence)	0.07%	0.35%	n/a	n/a	0.02%[54]	n/a	n/a	n/a	n/a
(*n*)	1	1	0	0		0	0	0	0
**Crohn's disease**	**3**	**5**	**2**	**3**		**n/a**	**n/a**	**n/a**	**n/a**
(prevalence)	0.60%	1.06%	0.36%	0.61%	0.20%[55]	n/a	n/a	n/a	n/a
(*n*)	9	3	2	4		0	0	0	0
**Ulcerative colitis**	**2**	**4**	**n/a**	**3**		**4**	**4**	**6**	**n/a**
(prevalence)	0.53%	1.06%	n/a	0.76%	0.24%[55]	0.85%	0.90%	1.50%	n/a
(*n*)	8	3	0	5		3	2	1	0
**Pernicious anemia**	**2**	**2**	**1**	**2**		**4**	**6**	**n/a**	**n/a**
(prevalence)	0.27%	0.35%	0.18%	0.30%	0.15%[50]	0.56%	0.90%	n/a	n/a
(*n*)	4	1	1	2		2	2	0	0
**Type I diabetes mellitus**	**2**	**n/a**	**5**	**1**		**2**	**2**	**n/a**	**n/a**
(prevalence)	0.40%	0.00%	0.90%	0.15%	0.19%[50]	0.28%	0.45%	n/a	n/a
(*n*)	6	0	5	1		1	1	0	0
**Hypothyroidism**	**2**	**2**	**2**	**2**		**1**	**1**	**0.4**	**0.6**
(prevalence)	15.27%	16.90%	16.40%	13.62%	6.90%[56]	7.34%	9.50%	3.00%	4.48%
(*n*)	229	48	91	90		26	21	2	3
**Sjögren's syndrome**	**1**	**1**	**2**	**1**		**n/a**	**n/a**	**n/a**	**n/a**
(prevalence)	0.47%	0.35%	0.54%	0.45%	0.32%[50]	n/a	n/a	n/a	n/a
(*n*)	7	1	3	3		0	0	0	0
**Celiac disease**	**1**	**n/a**	**1**	**1**		**n/a**	**n/a**	**n/a**	**n/a**
(prevalence)	0.47%	0.00%	0.54%	0.60%	0.83%[57]	n/a	n/a	n/a	n/a
(*n*)	7	0	3	4		0	0	0	0
**Ankylosing spondylitis/HLA B27**	**1**	**1**	**0.4**	**1**		**n/a**	**n/a**	**n/a**	**n/a**
(prevalence)	0.33%	0.35%	0.18%	0.45%	0.50%[53]	n/a	n/a	n/a	n/a
(*n*)	5	1	1	3		0	0	0	0
**Graves thyroiditis**	**1**	**1**	**1**	**1**		**n/a**	**n/a**	**n/a**	**n/a**
(prevalence)	1.60%	1.76%	1.80%	1.36%	2%[56]	n/a	n/a	n/a	n/a
(*n*)	24	5	10	10		0	0	0	0
**Rheumatoid arthritis**	**1**	**1**	**2**	**2**		**1**	**0.5**	**4**	**n/a**
(prevalence)	1.27%	0.70%	1.44%	1.36%	0.86%[50]	0.85%	0.45%	3.00%	n/a
(*n*)	19	2	8	9		3	1	2	0
**Psoriasis**	**0.8**	**1.2**	**1.1**	**0.4**		**1.5**	**2.1**	**1.0**	**n/a**
(prevalence)	1.13%	1.76%	1.62%	0.60%	1.5%[58]	2.26%	3.17%	1.50%	n/a
(*n*)	17	5	9	4		8	7	1	0
**Discoid lupus**	**0.3**	**n/a**	**0.2**	**1**		**n/a**	**n/a**	**n/a**	**n/a**
(prevalence)	0.27%	n/a	0.18%	0.45%	0.80%[59]	n/a	n/a	n/a	n/a
(*n*)	4	0	1	3		0	0	0	0
**Vitiligo**	**0.3**	**n/a**	**1**	**n/a**		**0.7**	**1**	**n/a**	**n/a**
(prevalence)	0.13%	n/a	0.36%	n/a	0.40%[50]	0.28%	0.45%	n/a	n/a
(*n*)	2	0	2	0		1	1	0	0
**Lichen sclerosus**	**0.2**	**1**	**n/a**	**n/a**		**1**	**2**	**n/a**	**n/a**
(prevalence)	0.07%	0.35%	n/a	n/a	0.30%[60]	0.28%	0.45%	n/a	n/a
(n)	1	1	0	0		1	1	0	0
**Lupoid hepatitis**	**n/a**	**n/a**	**n/a**	**n/a**		**14**	**23**	**n/a**	**n/a**
(prevalence)	n/a	n/a	n/a	n/a	0.02%[61]	0.28%	0.45%	n/a	n/a
(*n*)	0	0	0	0		1	1	0	0
**Polymyalgia rheumatica**	**n/a**	**n/a**	**n/a**	**n/a**		**1**	**0.5**	**n/a**	**4**
(prevalence)	n/a	n/a	n/a	n/a	1.00%[62]	1.13%	0.45%	n/a	4.48%
(*n*)	0	0	0	0		4	1	0	3

*Notes*: Each colored cell displays the estimated odds ratio, in bold, on top, its prevalence, in the middle, and numerical count on the bottom. Autoimmune diseases were rank ordered by the estimated odds ratio within the total MAC cohort. Disorders not observed in the MAC cohort were similarly ordered in the AUMC & MCJ. The color coding within each cell reflects the estimated odds ratio for that particular autoimmune condition: Red ≥ 20x or greater; tangerine/dark orange = 10–19x; light orange = 5–9x; yellow = 3–4x; green = 1–2x; blue < 1x; gray = none observed/not applicable. Both datasets revealed similar patterns of autoimmunity within total AD cohorts as well as across subcohorts.

Abbreviations: AD, Alzheimer's Disease; ANCA, antineutrophil cytoplasmic antibody; AUMC, Amsterdam University Medical Center; EOAD, early‐onset Alzheimer's disease; LOAD, late‐onset Alzheimer's disease; MAC, Memory and Aging Center; MCJ, Mayo Clinic, Jacksonville.

Bold numbers refer to the primary result (estimated odds ratio) and the results in regular formatting show the relative prevalence or raw counts of select autoimmune diseases.

## 4 DISCUSSION

In this study, we explored the demographic and health histories of early‐onset and non‐amnestic AD compared to LOAD and discovered that non–right‐handedness, learning disability, seizure, and autoimmune disease each influenced the age at onset and phenotypical targeting of AD. Using NACC, AUMC, and MCJ cohorts for validation, the total sample population studied exceeded 10,000 AD participants. While previous studies have individually linked these factors to AD susceptibility,^5^, ^7^, ^22^, ^23^ the scale and focus on interrelationships among factors here results in novel hypotheses explaining AD heterogeneity, offers opportunities to redefine diagnostic terms based on underlying biology, identifies potential therapeutic targets, and provides new theoretical frameworks for conceptualizing individualized neurodegenerative disease risk, which we discuss below.

Evaluating each factor in detail, non–right‐handedness rates were not only significantly elevated in EOAD compared to LOAD but were greater than the general population (15.5% vs. 10%, *p *< 0.001), with highest prevalence in PCA (Table S3), supporting hypotheses that neurodevelopmental differences are particularly relevant to non‐amnestic AD. Accordingly, we confirmed past observations between the domain of learning difference and AD phenotype as language and motor speech learning disabilities were highest in lvPPA (19.3%) and mathematical‐based learning disabilities were highest in PCA (18.6%).^5^, ^6^ Recent and remote seizure history, captured in the manner defined by the NACC, each displayed inverse associations with AD age at onset, in line with hypotheses that chronic hyperexcitability drives AD pathophysiology, and vice versa.^7^, ^24^ Within the MAC and external AUMC and MCJ validation cohorts, autoimmune conditions presented in a conspicuous gradient with the greatest number and degree of overrepresentation observed in non‐amnestic AD followed by EOAD and LOAD (Table 3). Moreover, of the seven autoimmune diseases present at rates > 10 times expected in the MAC cohort, six replicated within external AUMC and MCJ validation cohorts. Non‐amnestic AD possesses a higher degree of inflammatory microglial‐associated changes than amnestic AD,^25^ together suggesting that autoimmune disease may be more strongly associated with risk for focal‐cortical over hippocampal‐based AD.

Given the capricious origins behind the definition of EOAD (first symptoms < 65),^2^ as all novel factors were overrepresented in EOAD and typical risks factors in LOAD, we modeled the distributions of factors across the MAC cohort, which resulted in an age < 70 that maximized the distribution of differences between novel and typical AD risks factors (Figure 2). As the current EOAD/LOAD dichotomy lacks a clear biological basis, we evaluated the rates of these factors across gradients of age of onset. Novel factors displayed inverse linear relationships, such that differences presented between any two age ranges, even within respective designations of EOAD and LOAD, whereas typical AD risk factors better fit quadratic functions (Figure 3; and thus are less relevant to oldest old AD presentations, too, supporting past findings^26^). Combined, rather than maintaining a single EOAD/LOAD age divide, our data suggest that pathophysiological differences in AD present across three age‐defined epochs: early onset (< 70), typical onset (70‐85), and LOAD (> 85), though with improved access to early‐life education and decreases in cardiovascular disease burden, the divides between these categories may prove to be mutable, requiring periodic reevaluation.

Investigating the interrelationships among these factors, principal component analyses reduced the typical factors into a “vascular disease” factor, non–right‐handedness and learning disability into a putative “neurodevelopmental” factor, and seizure and autoimmune disease into a “neural environmental” factor. There is substantial precedence for these reductions, as increased prevalence of non–right‐handedness with learning disability has been well described,^27^, ^28^ and systemic chronic inflammation is known to instigate seizure activity.^29^ Moreover, prior observations within neurodegenerative disorders demonstrate that neurodevelopmental differences^5^, ^6^ and chronic neural environmental insults^8^, ^30^ hold unique status with regard to disease susceptibility. AD groups possessing both factors demonstrated exponentially greater reductions in age at onset than predicted for each alone (Figures 4 and S2), supporting a “two‐hit” model of neurodegenerative disease, in which structurally, developmentally vulnerable brain regions become susceptible to injury from sustained inflammation and focal neuronal hyperexcitability, facilitating focal neurodegenerative disease.

As previously observed, non‐amnestic AD presentations were disproportionally represented within EOAD,^3^ and as such, we performed analyses removing non‐amnestic AD from EOAD and LOAD comparisons, in which all prior differences survived (Table S2). Given the expected, observed differences in *APOE* ɛ4 rates between non‐amnestic and amnestic AD cohorts (Table S2), we investigated interactions between *APOE* ɛ4 status and novel factors (restricting these analyses to participants who possessed at least one novel factor). Here, *APOE* ɛ4 positivity provided additional reductions in age at onset only within amnestic AD presentations (Figure S1). Thus, depending on AD disease phenotype, the novel factors we detail demonstrated effects that were independent of, or synergistic with, *APOE* ɛ4 mechanisms of disease, highlighting their distinct relevance to AD risk modeling.

Differentiating non‐amnestic from amnestic AD highlighted the degree to which neurodevelopmental and neural environmental factors were overrepresented in non‐amnestic AD (Tables S2 and S3). Speculating on the origins of this enrichment, a genome‐wide association study of PCA identified three putative risk genes, all with neurodevelopmental functions.^31^ One candidate, *SEMA3C*, a member of the semaphorin gene family, also plays significant roles in autoimmune disease, including many identified in our AD collection.^32^ This dual function was first recognized in major histocompatibility complex class I proteins, which initially function in visual system radial glial migration before acquiring roles in antigen recognition (failures of which promote autoimmunity).^33^ An increasing number of genes exhibit similar dual roles,^34^ including AD‐related genes *TREM2* ^35^ and *APOE* ɛ4.^36^, ^37^ Accordingly, it has been proposed that polymorphisms in genes that first function in neurodevelopment and later act in maintaining cellular homeostasis, produce conditions in which developmental differences and later‐life susceptibility to neurodegenerative disease co‐occur, resulting in age‐dependent selective, focal attacks on developmentally vulnerable brain regions.^38^, ^39^ This would most parsimoniously account for the domain‐specific associations we observe between learning disability history and AD phenotype as well as the exponential decrease in age at onset witnessed in those individuals who had both neurodevelopmental and neural environmental factors (Figures 4 and S2), reflecting a “two‐in‐one” hit variation to the “two‐hit” model proposed above.

As the data for this study were largely derived from retrospective chart review of participants from tertiary referral centers, our results are prone to ascertainment bias and may not fully reflect the general AD population. Moreover, a large proportion of MAC and NACC data were collected prior to the advent and widespread use of AD biomarkers. Nonetheless, *n* = 273 of the MAC cohort had amyloid‐biomarker or pathology‐supported AD (meeting Core 1 AD staging criteria^40^), and analyses restricted to this group confirmed relationships between novel and typical factors (Table S4). Integration of new blood‐based amyloid biomarkers, increased use of tau PET and newer biofluid collections, and expected increases in our autopsy cohort will enhance scalability and facilitate correlative investigations between these factors with a broader range of neuropathologies. As the focus of this study was to investigate factors that alter AD age at onset and clinical presentation, it remains to be determined if and to what extent these factors confer risk of neurodegenerative disease. Additionally, in this initial investigation, we did not correct for multiple comparisons, as we had strong a priori hypotheses based on prior observations; however, moving forward we encourage integrating neurodevelopmental and neural environmental factors into population‐based studies of healthy aging and dementia cohorts and specifically incorporated our questionnaires on autoimmune disease and developmental history into the Longitudinal Early‐Onset Alzheimer's Disease Study (LEADS)^41^ to expedite this process. Given generational differences between EOAD and LOAD cohorts, we explored the possibility that societal attitudes toward developmental differences could compromise our findings. Mitigating concerns, rates of forced right‐handedness were actually higher in EOAD than LOAD (0.8%, *n* = 6/750 vs. 0.3%, *n* = 2/750) and learning disability history was likely equally underestimated across the entire cohort, as all MAC participants were born before 1970 and widespread recognition of developmental dyslexia did not occur until the 1970s.^42^ Regarding the possibility of systematic dropout from premature death of individuals suffering from autoimmune disease or seizure, prior to AD diagnosis, we note that autoimmune disease mortality rates are low (14.6 per million),^43^ while individuals with seizure diagnosed in the context of their AD diagnosis (and therefore would not be subject to concerns of systematic dropout) possessed the same inverse association with AD age at onset as those with remote seizure history. Whether the associations between these novel factors and AD age at onset reflect generalized phenomena relevant to each group (e.g., all seizure), or are specific to particular subpopulations within the broader class (e.g., focal vs. generalized vs. febrile seizures) remains uncertain, but we anticipate that future studies will be designed to capture the specific attributes within these novel factors that impact AD age at onset and phenotypical targeting.

Together, the findings from this study of > 10,000 clinically defined AD participants challenge current conceptions of AD risk beyond vascular disease, supporting a broader capture of health and demographic attributes, including neurodevelopmental and neural environmental factors. Insights from this study argue for a data‐driven re‐examination of current AD diagnostic definitions and nomenclature, introduce novel theories of AD susceptibility and phenotypic heterogeneity, and offer new opportunities for personalized AD risk modeling, enhancing disease prediction and intervention strategies.

## CONFLICT OF INTEREST STATEMENT

Zachary A. Miller reports no relevant disclosures. Rik Ossenkoppele has been an associate editor for *Alzheimer's Research & Therapy* since 2018. Neill R. Graff‐Radford is partially funded by the David Eisenberg Mayo Clinic Professorship. Isabel E. Allen, Wendy Shwe, Lynne Rosenberg, Dustin J. Olguin, Rahul S. Desikan, Yolande Pijnenburg, Emma Wolters, Rosa Rademakers, Daniel H. Geschwind, Joel H. Kramer, Howard J. Rosen, Katherine P. Rankin, Lea T. Grinberg, William W. Seeley, Virginia Sturm, David C. Perry report no relevant disclosures. Michael G. Erkkinen has received consultancy fees from Biogen. P. Monroe Butler contributed to this work during fellowship at the UCSF Memory and Aging Center and reports no relevant disclosures. Salvatore Spina reports receiving consulting fees from Acsel Health, Precision Xtract, and Techspert.io. Jennifer S. Yokoyama reports grants from the National Institute on Aging during the conduct of the study; grants from National Institute on Aging, Rainwater Charitable Foundation, Transposon Therapeutics, Alector, and the US Department of Defense; and other support from the Mary Oakley Foundation and French Foundation outside the submitted work. Wiesje van der Flier is a guest editor *Alzheimer Research Therapy* (series on SCD, 2018–2019); associate editor *Alzheimer Research Therapy* (2020–). Philip Scheltens has received consultancy fees (paid to the institution) from AC Immune, Alkermes, Alnylam, Alzheon, Anavex, Biogen, Brainstorm Cell, Cortexyme, Denali, EIP, ImmunoBrain Checkpoint, GemVax, Genentech, Green Valley, Novartis, Novo Nordisk, PeopleBio, Renew LLC, Roche; he is PI of studies with AC Immune, CogRx, FUJI‐ film/Toyama, IONIS, UCB, Vivoryon; he is a part‐time employee of Life Sciences Partners Amsterdam; and he serves on the board of the Brain Research Center. Bruce L. Miller reports serving on the Cambridge National Institute for Health Research Biomedical Research Centre advisory committee and its subunit, the Biomedical Research Unit in Dementia; serving as a board member for the American Brain Foundation; serving on John Douglas French Alzheimer's Foundation board of directors; serving on the Safely You board of directors; serving as a scientific director for the Tau Consortium; serving as a medical advisor for and receiving a grant from The Bluefield Project for Frontotemporal Dementia Research; serving as a consultant for Rainwater Charitable Foundation, Stanford Alzheimer's Disease Research Center, Buck Institute SAB, Larry L. Hillblom Foundation, University of Texas Center for Brain Health, University of Washington Alzheimer's Disease Research Center EAB, and Harvard University Alzheimer's Disease Research Center EAB; receiving royalties from Guilford Press, Cambridge University Press, Johns Hopkins Press, and Oxford University Press; serving as an editor for *Neurocase*; serving as a section editor for *Frontiers in Neurology*; and receiving grants P30 AG062422, P01 AG019724, R01 AG057234, and T32 AG023481 from the NIH. Gil D. Rabinovici reports grants from the National Institutes of Health, Alzheimer's Association, the American College of Radiology, Rainwater Charitable Foundation, Avid Radiopharmaceuticals, Eli Lilly, GE Healthcare, Life Molecular Imaging, and Genentech, as well as personal fees from Axon Neurosciences, Genentech, Johnson & Johnson, F. Hoffman–La Roche, and GE Healthcare outside the submitted work for service on scientific advisory boards (Axon Neurosciences, Eisai, Genentech, and F. Hoffman–La Roche) and a data safety monitoring board (Johnson & Johnson). Maria Luisa Gorno‐Tempini reports no relevant disclosures. Author disclosures are available in the supporting information.

## Supporting information



Supporting Information

Supporting Information

## Data Availability

All data used in this study are available for review upon formal request. As the institutional procedures in place at the time participants gave informed consent do not authorize open data sharing, all requests will need to undergo UCSF MAC regulated procedures including the submission of a materials transfer agreement. The requesting party will need to provide their name and affiliation as well as a brief description of their intended use of the data.
